# Preventing mental illness: closing the evidence-practice gap through workforce and services planning

**DOI:** 10.1186/s12913-015-0954-5

**Published:** 2015-07-24

**Authors:** Gareth Furber, Leonie Segal, Matthew Leach, Catherine Turnbull, Nicholas Procter, Mark Diamond, Stephanie Miller, Patrick McGorry

**Affiliations:** 1 grid.1026.50000000089945086Health Economics and Social Policy Group, School of Population Health, University of South Australia, Adelaide, Australia; 2 grid.1026.50000000089945086School of Nursing and Midwifery, Sansom Institute, University of South Australia, Adelaide, Australia; 3 Department for Health and Ageing, South Australia, Adelaide, Australia; 4 Australian Regional and Remote Community Services Ltd, Adelaide, Australia; 5 Survivors of Torture and Trauma Assistance and Rehabilitation Services, Adelaide, Australia; 6 grid.1008.9000000012179088XOrygen Youth Health Research Centre, Centre for Youth Mental Health, The University of Melbourne, Melbourne, Victoria Australia

**Keywords:** Mental illness, Prevention, Workforce planning, Health services planning, Health policy

## Abstract

**Background:**

Mental illness is prevalent across the globe and affects multiple aspects of life. Despite advances in treatment, there is little evidence that prevalence rates of mental illness are falling. While the prevention of cardiovascular disease and cancers are common in the policy dialogue and in service delivery, the prevention of mental illness remains a neglected area. There is accumulating evidence that mental illness is at least partially preventable, with increasing recognition that its antecedents are often found in infancy, childhood, adolescence and youth, creating multiple opportunities into young adulthood for prevention. Developing valid and reproducible methods for translating the evidence base in mental illness prevention into actionable policy recommendations is a crucial step in taking the prevention agenda forward.

**Method:**

Building on an aetiological model of adult mental illness that emphasizes the importance of intervening during infancy, childhood, adolescence and youth, we adapted a workforce and service planning framework, originally applied to diabetes care, to the analysis of the workforce and service structures required for best-practice prevention of mental illness.

**Results:**

The resulting framework consists of 6 steps that include identifying priority risk factors, profiling the population in terms of these risk factors to identify at-risk groups, matching these at-risk groups to best-practice interventions, translation of these interventions to competencies, translation of competencies to workforce and service estimates, and finally, exploring the policy implications of these workforce and services estimates. The framework outlines the specific tasks involved in translating the evidence-base in prevention, to clearly actionable workforce, service delivery and funding recommendations.

**Conclusions:**

The framework describes the means to deliver mental illness prevention that the literature indicates is achievable, and is the basis of an ongoing project to model the workforce and service structures required for mental illness prevention.

## Background

### The burden of mental illness

Mental illness is becoming a premier global health concern [1–8]. According to the World Health Organisation (WHO) World Mental Health Survey Initiative, the estimated projected lifetime risk of any mental disorder by the age of 75 years ranges from 18 % (PR China) to 55 % (United States), with 10 of 17 participating countries recording estimates of over 30 % [9]. Recently published global burden of disease estimates identify mental and substance use disorders as the leading cause, worldwide, of years lost to disability (YLDs) [10].

The effects of mental illness cover virtually every sphere of life. Studies in developed countries have documented that mental illness is associated with substantial premature mortality [11–18], unemployment and welfare dependency [19–21], homelessness [22–25], comorbid substance use and addiction [26–31], delinquency and detention [32–35], poor physical health [36–39], involvement in the child welfare system [40–43], risk-taking behaviour [44–47] and suicide [48]. The effects of mental illness are borne not only by the individual, but also family, friends, the wider community and the economy. The individual, social and economic burdens of mental illness are large, and comparable to that of physical illness [49–52]. Furthermore, the burden of mental illness is intergenerational, with parental mental disorders being a key risk factor for mental disorders in children [53].

### Treatment alone cannot address the burden

Despite advances in treatment options, increasing rates of treatment, and increased expenditure on mental health services [54, 55], the prevalence of mental illness and its impact on mortality has not improved in Australia over the last 20 years [56, 57]. This reflects a combination of limited effectiveness of treatments and only partial access to and uptake of treatment. A high proportion of individuals suffering from mental illness do not seek or receive adequate treatment. The World Health Organisation estimated that in 2001–2003, between 36 % and 50 % of serious cases of mental illness in developed countries, and between 76 % and 85 % in less-developed countries received no treatment in the previous 12 months [58]. Delays of several years between illness onset and first treatment are also common [59, 60]. Even when treatment is sought, US data suggests only 15 % of all individuals with serious mental illness receive minimally adequate treatment [61].

Poor adherence and non-response to treatments are additional challenges facing the management of mental illness [62, 63]. For example, a systematic review by Nose and colleagues [63] found that the weighted mean rate of non-adherence to treatment programs in people with psychosis was 26 %, while Souery and colleagues, reporting on prior meta-analyses, suggest that up to 50 % of individuals receiving treatment for depression could be classed as first-line non-responders [64]. Thus, even with ideal treatment coverage, limitations in the effectiveness of current treatments mean that 50–60 % of the burden of mental illness is estimated to be unavertable through treatment alone [65].

### Adult mental illness is at least partially preventable

Twenty years ago, the US Institute of Medicine (IOM) released a seminal report arguing for a greater focus on the prevention of mental illness and for significant increases to US funding for prevention research and implementation infrastructure [66, 67]. The authors proposed a tight definition of prevention (i.e. interventions occurring prior to onset of illness) to distinguish prevention from treatment and maintenance, and argued for a significant boost to prevention funding in both research and intervention. In support of this, the IOM report highlighted the demonstrated value of the classic risk reduction model, in which the mechanisms underlying the development of illness are disrupted through targeting modifiable risk and protective factors along the causal pathway. The authors argued through a review of the risk factor and intervention literature for five mental disorders - conduct disorder, depressive disorder, alcohol abuse and dependence, schizophrenia and Alzheimer’s disease - that there was already sufficient evidence to intervene on a range of risk factors, such as parental mental illness, families with poor parental interactions, and cases of child maltreatment.

Since the release of the IOM report, evidence that mental illness is at least partially preventable has continued to accumulate. For example, it has been established that the majority of adult mental illness starts in childhood, adolescence or youth. Kessler and colleagues, using data from the WHO’s World Mental Health Survey and other epidemiological surveys [68], concluded that “roughly half of all lifetime mental disorders in most studies start by the mid‐teens and three‐fourths by the mid‐20s”. Other longitudinal studies have demonstrated that almost eighty percent of adult disorders can be reframed as extensions of juvenile disorders prior to the age of 18 years [69]. Consistent with this, child and adolescent emotional and behavioural disorders are consistent and strong predictors of adult psychosocial functioning [70–74].

Specific risk factors in childhood, adolescence and youth are particularly powerful predictors of poor adult mental health outcomes. Data from the US Adverse Childhood Experiences study [ACE] have demonstrated just how powerful childhood determinants are as predictors of adult mental illness. Published ACE analyses report that 35–40 % of the burden of depression [75], 56-64 % of the burden of drug problems [76], 67–80 % of the burden of suicide attempts [77] and 55 % of the burden of early alcohol use [78] could be attributed to the exposure to one or more adverse childhood experiences such as abuse, neglect, parental mental illness, substance abuse, incarceration, divorce, or family violence. ACE studies have also revealed a dose–response relationship between level of risk factor exposure and adult mental illness, demonstrating the cumulative effect of these adverse experiences during the formative years of life.

Multiple other risk factors, clustered in childhood, adolescence and youth, have also been found to predict adult mental illness. For example, Fryers and Brugha reviewed the literature on childhood and adolescent determinants of adult mental illness, from which they identified multiple risk factors falling within the domains of psychological disturbance, genetic influences, neurological deviance, neuroticism, behaviour, school performance, adversity, child abuse or neglect, parenting and parent–child relationships, and disrupted and dysfunctional families [79]. Melchior and colleagues, using data from a longitudinal study of New Zealand children, demonstrated that the well-established relationship between childhood socioeconomic status (SES) and adult health reflected a child’s level of exposure to the types of risk factors indicated above [80].

The mechanisms through which these factors in childhood exert their influence are also being uncovered. For example, we know that “adverse childhood experiences are associated with enduring changes in the nervous, endocrine, and immune systems. These changes are already observable in childhood years and remain apparent in adult life” [81].

Most importantly, there is evidence that intervening during childhood, adolescence and youth to prevent mental disorders is possible and potentially cost-effective. For example, Sandler and colleagues’ mega-analysis of 48 different meta-analyses of prevention programs [82] identified many effective programs targeting children and adolescents, with demonstrated impacts on depression, anxiety, aggression, antisocial behaviour, violence and substance misuse. From an economic perspective, Knapp and colleagues reported on the potential returns on investment of a range of mental illness prevention and health promotion interventions in the UK. They found the total (short, medium and long-term) returns on programs delivered in childhood, adolescence and youth to be large: up to 83 times the cost of the intervention in the case of programs aimed at preventing conduct disorder [83].

In 2009, the IOM released a follow-up report on mental illness prevention in which the argument for prevention was renewed and expanded with a specific focus on young people [84]:

*“Several decades of research have shown that the promise and potential lifetime benefits of preventing mental, emotional, and behavioural (MEB) disorders are greatest by focusing on young people and that early interventions can be effective in delaying or preventing the onset of such disorders”* [84].

The authors of the follow-up report highlighted significant advances in knowledge since the 1994 report, particularly in the understanding of the developmental origins of mental illness and the demonstrated effectiveness of school and family-based prevention programs targeting substance abuse, conduct disorder, antisocial behaviour, aggression, maltreatment and depression. The authors argued that despite a growing evidence base there was still an insufficient focus on prevention in practice and they urged researchers and policy makers to make the prevention of mental, emotional and behavioural disorders in young people a national priority.

The core sentiments of these highly influential reports, namely that prevention of mental illness is *imperative, achievable and best targeted at young people*, continue to be echoed in multiple prevention reviews and commentaries [85–90], including the 2012 European Psychiatric Association’s guidelines for prevention [85]. In Australia, prevention of mental illness has featured prominently in mental health policy since the 1992 National Mental Health Policy document [91]. However, despite strong positions from high-profile national and international health agencies on the value of prevention in mental illness, this has not translated into widespread population-level mental illness prevention initiatives or research funding in Australia [92]. The policy dialogue around chronic disease prevention continues to focus on cardio-metabolic illness and preventable cancers, with lifestyle behaviours or biological risk markers as intervention targets [93].

### Reasons for a lack of progress

Amongst recommendations for further research to address these aforementioned issues, Jacka and colleagues identified knowledge-translation research as a priority for the field, arguing that:

*“In order to optimally engage the key agencies to ensure the translation of such research findings into informed public health policy, research needs to be targeted for use by decision makers and evidence presented in a form that is most useful for end users”* [94]*.*

Such knowledge-translation research is difficult and has a poor history of uptake. A systematic review of the use of research evidence in public health decision making processes found a “gulf between decision makers and researchers” in terms of the types of evidence generated in traditional research, versus the evidence required to guide policy development. It was recommended that more research target the needs of decision makers [95].

In an attempt to provide a guiding framework for policy translation in mental health, Whiteford and colleagues have described three important stages in translating evidence into policy in mental health system improvement [96]. The first stage involves (a) quantification of the disease burden (i.e. morbidity, mortality, economic and social impacts) and (b) describing the interventions that can reduce disease burden. Much of the policy-relevant research in mental illness prevention has been focused at this stage on exploring disease burden; reviewing available interventions from a policy perspective [97–99]; and to a lesser extent, conducting priority setting [100, 101] in which the relative performance (cost-effectiveness) of alternative intervention options are formally compared, to determine which interventions should be given priority. The second stage involves the organisation of identified interventions into a service delivery framework. In stage three, specific changes to policy are made to support implementation of the recommended service delivery frameworks.

To date, there have been no known attempts to translate evidence into workforce and service delivery frameworks for the prevention of mental illness, although we do acknowledge workforce studies in relation to treatment [102]. In recognising this need, the Australian Government in 2009 expressed its intention in the Fourth National Mental Health Plan [103] to develop a National Mental Health Service Planning Framework (NMHSPF). The objective of the NMHSPF project was to use evidence-based guidelines and epidemiological data to “estimate the range and quantity of mental health care required by our population and the resources required to provide it”, including scope to explore resources for *promotion and prevention*. However, five years on, there have been no reported outcomes from the NMHSPF project. Responding to the need for workforce relevant evidence, we present the first known paper outlining a description of a needs-based workforce and service modelling framework and its potential application to the prevention of mental illness.

### Introduction to needs-based workforce modelling and services planning

Needs-based workforce modelling and services planning is a type of evidence-informed health workforce planning. Health workforce planning refers to activities undertaken to ensure that an appropriate health workforce exists to meet health care needs [104]. Health workforce planning in principle incorporates two broad tasks: (a) estimating health workforce requirements based on an understanding of supply and approximations of demand to estimate the mismatch between these, and (b) implementing policy interventions to address shortfalls (or predicted oversupply) [104].

Evidence-based approaches to health workforce planning are surprisingly rare [105]. Workforce planning commonly rests on clinician-population ratios [105] that reflect historic patterns, pragmatic responses to service budgets [106], control of supply by professional groups [106], or demand-based approaches that observe the gap between expressed demand and supply. The first two approaches are essentially arbitrary and disconnected from demand, while the expressed demand approach is compromised by the characteristics of the market for health care, which mean that expressed demand (patient requests for services) will be distorted [107]. If supply is known to be limited (and say because of funding, unable to respond to demand), many in need will simply not seek services, and/or very restrictive eligibility criteria will be imposed. This means the observable market signals are simply inaccurate.

Needs-based frameworks offer an evidence-based approach to workforce planning that put estimation of need at the centre. The primary challenges of a needs-based framework are to develop a model that can reflect the complexity of the community and their health care needs, that is capable of translating those needs into clinical care requirements (based on competencies), and that can assimilate a complex evidence base in a way that is rigorous, transparent and tractable. The aim of needs-based health workforce planning is to estimate the workforce team (skill mix and staffing level) required to support best**-**practice care for one or more health conditions in a defined regional population. Needs-based frameworks provide a systematic way of describing what is required to reduce the gap between clinical care or service delivery and best practice.

There is a small health workforce planning literature that takes a needs-based approach, representing a considerable advance on previous models [108–116]. Birch and colleagues [113] have developed a sophisticated analytical framework for use in needs-based human resources planning, to model the impact on the health workforce of assumptions about provider supply and provider requirements, including estimates of need. In the mental health sphere, Andrews and colleagues [102] created a detailed needs-based, costed, stepped-care model for adult mental health treatment services. The model builds on estimates of the number of adults with specific mental illnesses, the available treatments, and the staff and facilities required to treat each condition. Bruckner and colleagues [109] used epidemiological data on the prevalence of adult and paediatric disorders, and assumptions regarding treatment coverage and service delivery to estimate the shortfall in mental health service providers in 58 low and middle income countries. Burke and colleagues [114] used staffing data, current service use patterns and national survey data to estimate the number of patients likely to need behavioural health care in US public health centres. They estimated the number of visits likely needed by health centre patients annually, and the number of full-time equivalent providers needed to serve them. Finally Hosie and colleagues [117] modelled the impact of a modest increase in demand for mental health services, based on an increased number of those with existing disorders seeking help, on the mental health budget. A common limitation of these models is an oversimplified profile of the population requiring care, which misses the complexity and comorbidity of mental illness presentations and the associated care requirements in the population.

Segal and colleagues [118–120] have published a needs-based workforce planning framework for estimating the workforce team (skill mix and staffing level) to support the delivery of best-practice primary and community care in chronic disease for a regional population. The framework recognises that the delivery of best-practice care requires a health workforce with an appropriate set of competencies in order to support an uncompromised model of delivering best-practice care. This underpins development of a workforce strategy. The framework takes a geographic region as the planning frame and combines data about the health needs of the regional population (e.g. prevalence of disease and other characteristics pertinent to care needs or risk factor status) with best-practice guidelines and interventions to estimate the clinical skill requirements and competencies for the region. The translation of these skill requirements into workforce or service structures is then modelled, incorporating various assumptions about the occupation group(s) best suited to deliver the identified competencies. As the model is competency-based, it has considerable flexibility, allowing for the generation of new or expanded clinical roles if indicated. These workforce or service structures can then be compared to current service delivery to define the gaps or surpluses in current practice. The results of applying the framework can be used to inform service delivery as well as a workforce supply strategy including education and training requirements.

In previous work, the framework was successfully applied to diabetes [120] to define the primary care team required to support best-practice diabetes care. Starting with the core diabetes types (e.g. Type 1, Type 2, gestational), the project utilised the current scientific literature, clinical practice guidelines and clinical advisory groups to (a) develop detailed clinical profiles of different diabetes presentations including the common complications and complicating factors influencing care; (b) identify the best-practice management of these presentations; (c) translate these protocols into clearly defined competencies; and (d) model and cost different workforce and service structures required to deliver these competencies to the estimated number of people with diabetes in Australia. Using the framework it was identified that primary and community care teams required a wider range of competencies in the treatment of diabetes than typically described. This included access to care teams with the competencies to manage the psychosocial needs of the patient as well as their medical needs, which reflected the diversity of presentations and multiple complicating and enabling factors in diabetes patients.

## Method

### Application of needs-based workforce modelling and service planning to mental disorder prevention

#### Goal

The goal of needs-based workforce modelling in mental illness prevention is to estimate the workforce and service structures required for a given regional population to effectively address core risk factors in infancy, childhood, adolescence and youth (‘young people’) that are implicated in the development of mental illness in adults.

### Underlying premises and definitions

Adult mental illness is defined by disorders listed in the Diagnostic and Statistical Manual of Mental Disorders (DSM-IV) [121] and the International Classification of Diseases (ICD-10) [122]. Rather than focus on the prevention of a single disorder (e.g. depression), our approach to workforce modelling in mental health is to consider multiple disorders simultaneously. This reflects the existence of a set of risk factors that feature in the development of multiple mental illnesses. Preventing adult mental illness is operationalised numerically as reductions in incidence (new cases) of diagnosed mental illness, and prevalence (percentage) of the population with diagnosed mental illness.

**Fig. 1:**
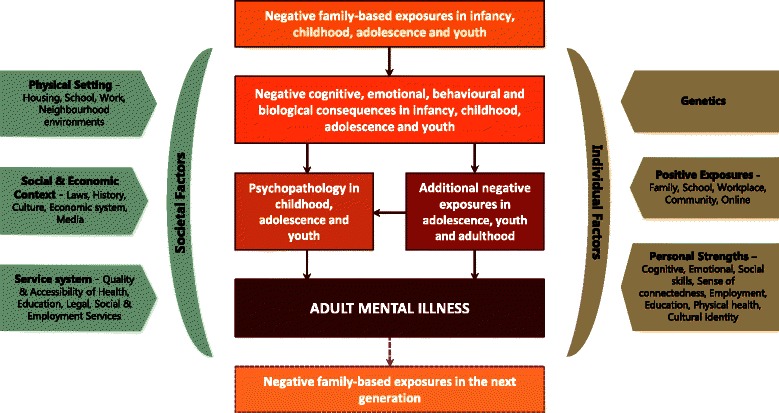
OrigAMI – Origins of Adult Mental Illness

Our conceptualisation of the development of adult mental illness is captured in Fig. 1. The Origins of Adult Mental Illness (OrigAMI) model is informed by an extensive review of the literature, the authors’ clinical work with children, adolescents and youth with mental illness, and the authors’ clinical and theoretical work in child maltreatment [123–125]. The model describes a central pathway where negative family-based exposures, such as abuse, neglect, family violence, poverty and parental mental illness, impact negatively on a child’s cognitive, emotional, behavioural and biological development, placing the child at increased risk of psychopathology, and increased risk of additional negative exposures such as bullying. These underlying vulnerabilities, some of which are present from birth (e.g. low birth weight due to intrauterine exposure to drugs) can greatly increase the risk of developing adult mental illness, especially if they accumulate over childhood, adolescence and into young adulthood. This central pathway is subject to multiple moderating influences. At the individual level, these include genetics, positive exposures (e.g. supportive teacher) and personal strengths (e.g. resilience, coping style). At the societal level, these include the quality of the local environment in which the individual lives, the quality and availability of health and social services, and the culture that the individual is exposed to. The model also captures the intergenerational nature of mental illness, in which adults become parents and their mental illness becomes a driver of negative family-based exposures in the next generation.

Importantly, the model emphasises that the prevention of adult mental illness is achieved through a focus on the preceding phases of life: infancy, childhood, adolescence and youth (referred henceforth as “in young people”). The logic of the model is that reducing young people’s exposure to negative family-based factors such as physical and sexual abuse, neglect, and domestic violence, and identifying and treating young people who suffer the negative consequences of these exposures, should be the key focus of adult mental illness prevention activities.

We understand from the literature that not all exposures and consequences (referred to collectively as risk factors) are of equal importance in the aetiology of adult mental illness. We also recognise that it may not be feasible to address all identified risk factors in a single workforce and services model. Thus, one of the critical steps in workforce and services planning in mental illness prevention is to prioritise risk factors in order to identify significant prevention “opportunities” [67]. These opportunities are defined as exposures or consequences (or clusters of exposures and consequences) whose modification is predicted to have significant effects on adult mental illness. This process of prioritisation is built into the workforce model and is described in Step 1 below.

We also recognise, based on the moderating influences described in the model, that the modification of these risk factors might be achieved from programs across diverse portfolios (e.g. health, education, employment, social services) and at different stages of the lifespan from conception to youth. Thus, a wide net must be cast when scoping for best-practice programs in Step 3 (described below).

Finally, we approach the workforce and services modelling from a perspective of delivering an uncompromised and ideal workforce and services model that is not necessarily tied to existing conceptualisations of workforce, professions, skill mix, service structures or funding. In this respect, the recommendations arising from the workforce and services planning activity might require considerable structural, funding or other reforms for their implementation. This approach includes a definition of ‘workforce’ that is broader than just those professions commonly associated with mental health (e.g. psychologists, psychiatrists, social workers, mental health nurses). It encompasses workers across different portfolios (e.g. education, employment), from other occupations (e.g. youth workers, teachers), and with varying levels of training, from lay-people (e.g. volunteers, neighbours, family members) and peer workers, through to specialised mental health professionals. This approach also allows for significant creativity in proposing new service structures, funding models, professional designations, and programs. It also allows for consideration of new training and credentialing arrangements to create new professional groups.

### The steps and tasks of needs-based workforce modelling and service planning in mental disorder prevention

**Fig. 2:**
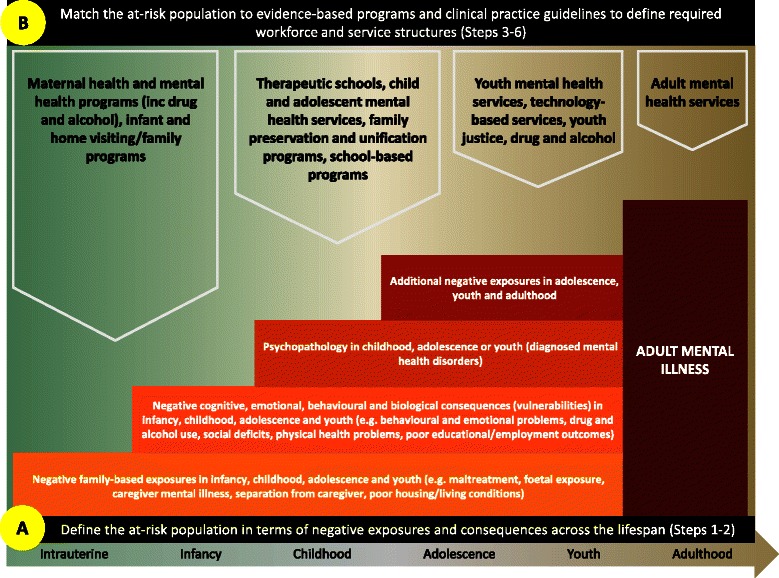
Workforce planning framework for mental illness prevention

Segal’s workforce planning framework for mental illness prevention (Fig. 2) provides a logical and repeatable process for moving from the defining of the at-risk population in terms of negative exposures and consequences across the lifespan (A), to defining the workforce and service structures required to deliver best-practice interventions with these at-risk groups (B). The six steps of this framework are described below. Readers interested in seeing how some of these steps were applied to diabetes care are directed towards the model application published elsewhere [120].

#### Step 1 – prioritising risk factors

The aim of Step 1 is to focus the workforce and services model around risk factors most relevant to reducing adult mental illness. Guided by the aetiological model (Fig. 1), it first involves identifying systematic reviews and meta-analyses of studies reporting on risk factors for adult mental illness. The reason for drawing on reviews is the sheer volume of studies that have reported on risk factors for mental illness. For example, a recent review published by Fryers and Brugha in 2013 [79], which limited its scope to longitudinal studies of childhood determinants of adult mental illness, found a large number of risk factors, but these risk factors collapsed into a relatively small number of clusters (mental, emotional and behavioural disorders, genetics, neurological deviance or damage, childhood adversity including abuse and neglect, family environment including parenting and parental mental illness, personality factors, and school performance/achievement).

The risk factors identified in these studies are then prioritised using criteria summarised in Table 1. The entry criterion is that the risk factor is modifiable. Other criteria are concerned with (i) quantitative importance, related to prevalence of the risk factor, and/or the strength of the relationship between the risk factor and adult mental illness; (ii) quality of evidence that the risk factor is a critical part of the causal pathway for the development of mental illness, established by a clearly defined causal mechanism and empirical evidence supportive of a causal relationship (i.e. temporality, dose–response); and (iii) the practicality of identifying the risk factor in the population. A desirable, but not necessary criterion is that there are interventions that have been shown to modify the risk factor. This is because we do not want to be restricted in identifying potent modifiable risk factors by the failure to build an evidence-base for modifying those risk factors. Taken together, these criteria help identify the set of risk factors that are responsible for a significant proportion of the preventable burden of adult mental illness. This process is analogous to methods employed by the WHO to prioritise risk factors for global prevention interventions [126].

**Table 1:** Criteria for identifying priority risk factors from risk factor literature

	Description
Entry criteria	
▪ Modifiability	The risk factor is, at least in theory, modifiable
Other necessary criteria	
▪ The relationship between the risk factor and adult mental illness is causal	*Established mechanism of action:* There are clear mechanisms by which the risk factor influences outcomes (e.g. established biological pathways)
	*Temporality*: The risk or protective factor precedes the outcome.
	*Dose–response*: Poorer mental illness outcomes associated with increased exposure to risk factor
▪ Size of effect	*Strength and Consistency*: Cross-sectional and longitudinal studies have demonstrated that the risk or protective factor is a strong and consistent predictor of adult mental illness outcomes.
	*Prevalence of risk factor:* the more common the risk factor the greater will be its contribution to incidence and burden (all else equal)
	*Multifinality*: The risk factor features in the aetiology of multiple mental illness outcomes (e.g. different disorders) and therefore its modification may influence multiple outcomes.
	*Health inequalities*: the risk factor is found to be unjustly distributed in certain population groups.
▪ Identifiability	The risk factor can be identified in the population through screening and surveillance.
Desirable criteria	
▪ Intervention opportunities	*Evidence base for interventions*: There are efficacious, effective and/or cost-effective interventions that modify the risk factor.
	*Clustering:* The risk factor clusters with other known risk factors for the outcome, suggesting interventions may be able to target multiple risk factors at the same time.

The selected risk factors are then screened by an expert advisory panel consisting of clinicians, service providers, academics and consumers. The use of such a panel is to ensure that the selected risk factors are comprehensive but not over-defined. This panel, of up to 20 or so individuals, is re-engaged throughout the subsequent stages to offer comment and advice on other aspects of the process, such as reviewing clinical service options and competencies attached to best practice. A separate consumer engagement strategy can be utilised if considered valuable.

#### Step 2 – profiling the at-risk population

The goal of Step 2 is to measure the distribution of the prioritised risk factors in the population; that is, to estimate the number at risk, in the defined risk categories. For example, mental, emotional and behavioural disorders (MEBD) in children, adolescents and youth are a significant risk factor for adult mental illness [70–74]. In Step 2, we determine how many children, adolescents and young people (within a specified population) have such disorders and how they are distributed demographically (e.g. age, gender, education, ethnicity or SES) and type of mental, emotional or behavioural problem (e.g. anxiety, depression, schizophrenia). The purpose of this detailed profiling is to (a) quantify the number of people in the population affected by selected risk factors, and (b) support improved matching of interventions/services to those at risk, by understanding how those risk factors are distributed.

Step 2 primarily involves a review of regionally representative observational data (longitudinal and cross-sectional) to understand the prevalence and distribution of the selected risk factors. Example data sets relevant to mental illness prevention in Australia include the National Survey of Mental Health and Wellbeing [127] and the Longitudinal Study of Australian Children [128]. Analyses of these data sets is supplemented by consultation with the expert advisory panel who identify additional sources of data regarding risk factor prevalence and highlight risk groups (those with higher prevalence of risk factors) who may not be well captured in observational data sets. Examples of such risk groups include refugees and young people in the justice system.

#### Step 3 – matching population needs to best practice

In Step 3, the risk factors and risk groups identified in Steps 1 and 2 are matched to relevant clinical practice guidelines and evidence-based programs with demonstrated impacts on both the risk factor and progression to mental, emotional or behavioural disorders and adult mental illness. As with Step 2, this process includes both a review of the relevant literature, starting with systematic reviews and meta-analyses, and the use of the expert advisory panel to identify best-practice programs to and provide guidance on how comorbidities and contextual factors could be managed in practice. Programs identified at this stage might come from diverse portfolios and be targeted at different stages of the lifespan. The output of Step 3 is a set of clinical practice guidelines and/or evidence-based programs that are available to address the identified risk factors and at-risk groups.

#### Step 4 – translation of best practice to competencies

In Step 4, the project team work closely with clinicians from the expert advisory panel to dissect each of the selected evidence-based programs and guidelines into a set of actionable tasks (competencies), which represent how that program would be implemented in practice. Clinicians are called on individually, or in small groups, based on having relevant experience with the program/guidelines being analysed.

Each competency is expressed in hours per annum per target individual (or family, in the case of family-based programs). For example, a competency statement describing the implementation of a drug treatment guideline for attention deficit hyperactivity disorder (ADHD) would include individual competencies relating to pre-drug assessment, titration, and monitoring, as well as an estimate of the time required per annum to complete these competencies for a single child. Competency statements are created for each of the selected programs or guidelines derived from Step 3.

As part of the competency process, clinicians also consider and make recommendations on what professions are best qualified to carry out the competency. In cases where competencies can potentially be carried out by different professionals, the budget implications of using different professionals can be explored in Step 5. The clinical advisory group can also suggest new professional classifications if the competencies suggest a unique combination of skills that are not well captured by an existing profession. By the conclusion of Step 4, each program and guideline has a corresponding competency statement.

#### Step 5 – translation of competencies to workforce and services estimates

The hours of each competency required to deliver best practice care described in Step 4 are combined with the estimated prevalence rates of the sub-groups (from Step 2) to estimate the total hours by competency. Taking from Step 4 the estimated clinician time, by competency, required to deliver the identified best practice programs to one person over a year, we extrapolate to the total number of hours at the population level using the risk factor prevalence estimates from Step 2. Survey-based estimates of hours allocated to direct clinical service delivery per clinician per year are used to translate into full-time equivalent (FTE) workforce estimates by competency.

Step 5 also includes exploration of different workforce and service structures and their impact on the cost-efficiency of delivering the identified competencies. This includes the cost implications of (a) using different professionals or workers to carry out competencies, and (b) combining programs into defined services such as specialised child and adolescent mental health services for the management of mental, emotional and behavioural disorders.

#### Step 6 – exploring policy implications of workforce and services estimates

Step 6 comprises multiple activities that can be undertaken to explore the policy implications of the different workforce and service structures proposed in Step 5. These activities are summarised in Table 2. This step involves further stakeholder consultation. For example, in our work we have established a strategic partnership with the State Department of Health to assist in the translation of the results into practice.

**Table 2:** Exploring the policy implications of workforce and service modelling

Policy question	Description
What potential impact will implementing the workforce and service structures have on incidence and prevalence rates of adult mental illness?	Assuming the workforce and service structures from Step 4 are implemented at full fidelity and reach all relevant individuals/families, the theoretical impact on rates of adult mental illness are estimated using population attributable fractions (PAF). PAF provide an estimate of the burden of mental illness that is attributable to an individual or group of risk factors.
What are the potential cost-savings of implementing the proposed workforce and service structures?	In this activity we use estimates of the societal costs of mental illness to determine the potential savings of reducing rates of adult mental illness. This can then be compared to the costs of implementing the prevention programs.
What are the shortfalls and surpluses in terms of professionals available to deliver the proposed workforce and service structures?	This activity uses Australian Bureau of Statistics (ABS) Population Census data items on workforce, qualifications, place of work, industry, employment status, age, gender and hours of work to estimate the current and potential health workforce in defined regions. This can then be compared with the workforce estimates from Step 4 to identify major areas of imbalance.
Are current prevention activities in Australia consistent with the evidence-base?	In conjunction with a review of current Australian prevention activities, we can compare against those recommended from the modelling to identify gaps in best-practice prevention provision.
What resources will be required to implement the proposed workforce and service structures? How should those resources be distributed?	Explore the cost implications of alternative delivery models and workforce mixes. This will be informed by standard fees or training costs for each occupation. The impact on cost, of delivery characteristics such as occupational mix, mode of service delivery (individual/group, face-to-face/internet/phone, team-based or single clinician) will be explored. These analyses also include questions of whether services should be state or nationally funded.
How does current spending on prevention compare to that required to implement the proposed workforce and service structures?	Survey the human services system to determine how much is currently being spent at a state and commonwealth level on mental illness prevention. Compare this funding to that required to deliver the proposed workforce and service structures to determine resources shortfall.
What are the education and training implications of implementing the proposed workforce and service structures? What new professional classifications might be needed to be created to deliver the proposed services?	Use the analysis of competencies in Step 3 to determine what additional or specialised training might be required to prepare health, education or social welfare service professionals to staff the proposed workforce or service structures. In addition, explore new professional classifications to capture the unique set of skills required to intervene with children, adolescents and youth at risk of mental illness.
How do we translate the proposed workforce and services structures to local level changes?	In this activity, we collaborate with local health, education and social welfare service administrators to explore options for changing funding and delivery model to support implementation of the proposed workforce and service structures.
What may be the additional flow-on effects of implementing the workforce and service structures recommended in the project?	Quantifying the potential impacts on other domains. For example, some mental health programs may have impacts beyond mental illness (e.g. physical health, family quality of life, education attendance, work attendance).

## Results and discussion

Chronic illnesses in adulthood, including mental illness are increasingly being viewed through a developmental lens [129, 130]. Through this lens, experiences starting in-utero and continuing through infancy, childhood, adolescence and youth are crucial in laying the foundations for long-term health and wellness. Decades of evidence in mental illness prevention research supports this view, with multiple different programs supporting young people demonstrating capacity to reduce risk for future mental health problems [82]. The concept of supporting young people and families to build a healthy adult population is central to mental illness prevention.

Our observation is that the prevention dialogue in Australian health is largely focused around cardiovascular disease, cancer and obesity. It is common to hear people speak of risk of heart attack, or risk of developing cancer based on genetic, behavioural or environmental exposures. It is also expected that general practitioners will speak with patients about their risk for these conditions, and make recommendations for lifestyle changes or recommend preventive treatments. It is far less common, however, to hear people talk about the risk of developing mental illness, or for health professionals to inform those who might be at risk, based on presenting risk factors and recommend steps that might be taken to reduce risk. From a prevention perspective, the stigma associated with mental illness may be a significant barrier to intervention.

We are arguing that there is an opportunity to address this issue at the population level, through well-targeted mental health informed services delivered at key junctions over the lifespan. Similar to Hosie and colleagues [117] who argued for reforms to the mental health system to avert unsustainable growth in demand for treatment, we believe there are multiple opportunities from before birth into early adulthood to reduce people’s risk of developing mental illness. A number of areas of research have furthered our understanding of the key modifiable risk factors for adult mental illness (such as adverse childhood experiences and child/adolescent emotional and behavioural disorders) and at the same time there is a growing body of evidence-based interventions to address these factors.

However, there remains a gap in operationalising this evidence, which requires appropriate workforce and service structures to deliver evidence-based interventions at a population level. Determining the workforce and service structures and the associated resource implications needed to deliver a preventive mental health agenda is required by policy makers to take this agenda forward, but there has been limited work in this area. When Pirkis et al. [131] reviewed 32 mental health plans from five developed countries that outlined core mental health services required, they found only 4 cited specific resource targets based on some evidence-based rationale.

The needs-based workforce and services planning framework described in this paper offers a preliminary way of translating the evidence-base in mental health prevention to specific recommendations regarding workforce structure and skill mix. Because of its flexibility, the framework can be adapted to different regions (using local population data), different illness types (targeting different risk factors), and local intervention capacities (local adaptations of best practice). We believe this work complements the broader advocacy work being done by groups like the Alliance for Prevention of Mental Disorders [132] in Australia and provides a way to develop “evidence-based models of care” [133] in which service structures are tightly linked to current best-practice evidence.

## Conclusion

The prevention of mental illness is imperative, achievable and best targeted at young people. As our understanding of the aetiology of mental illness improves, so should our capacity to translate that evidence into population-level initiatives to prevent mental illness. Needs-based workforce planning frameworks offer methods to achieve this by identifying core risk factor targets and translating best-practice management of those risk factors into workforce and service structures for a defined regional population. In this paper we have described the process of needs-based workforce planning in mental illness prevention. We plan to present the findings from the implementation of this work within the context of Australian health services in the near future.

## Abbreviations

WHO: World Health Organisation
IOM: Institute of Medicine
MEBD: Mental, emotional and behavioural disorders
NMHSPF: National Mental Health Service Planning Framework
DSM-IV: Diagnostic and Statistical Manual of Mental Disorders
ICD (10): International Classification of Diseases Version 10
CAMHS: Child and Adolescent Mental Health Services
FTE: Full time Equivalent
